# Lactoferrin Supplementation in Preventing and Protecting from SARS-CoV-2 Infection: Is There Any Role in General and Special Populations? An Updated Review of Literature

**DOI:** 10.3390/ijms251910248

**Published:** 2024-09-24

**Authors:** Paolo Manzoni, Alessandro Messina, Chiara Germano, Simonetta Picone, Bianca Masturzo, Pier Paolo Sainaghi, Daniele Sola, Manuela Rizzi

**Affiliations:** 1 Department of Maternal, Neonatal and Infant Medicine, University Hospital “Degli Infermi”, 13875 Ponderano, Italybianca.masturzo@aslbi.piemonte.it (B.M.); 2 School of Medicine, University of Turin, 10124 Turin, Italy; alessandro.messina@hotmail.it; 3 Sant’Anna Hospital, Department of Surgical Sciences, University of Turin, 10126 Turin, Italy; 4 Neonatology and Neonatal Intensive Care Unit, Policlinico Casilino, 00169 Rome, Italy; 5 Department of Translational Medicine (DiMeT), Università del Piemonte Orientale (UPO), 28100 Novara, Italy; 6 IRCAD (Interdisciplinary Research Center of Autoimmune Diseases), Università del Piemonte Orientale (UPO), 28100 Novara, Italy; 7 Laboratory of Metabolic Research, IRCCS Istituto Auxologico Italiano, S. Giuseppe Hospital, 28824 Piancavallo, Italy; 8 Department of Health Sciences (DiSS), Università del Piemonte Orientale (UPO), 28100 Novara, Italy

**Keywords:** COVID-19, SARS-CoV-2, lactoferrin, prevention, treatment, outcome, infants, pregnancy

## Abstract

At the beginning of the pandemic, SARS-CoV-2 infection represented a great medical burden worldwide, as targeted and effective therapeutic options were lacking. This resulted in the revival of existing molecules and the increasing popularity of over-the-counter nutritional supplements. Among the latter, lactoferrin has been investigated as an adjuvant in COVID-19 therapy with conflicting results, mainly depending on different study designs. Considering that lactoferrin is one of the main components of human breast milk with anti-microbial and anti-inflammatory activity, it is conceivable that such bioactive molecule could be effective in supporting anti-SARS-CoV-2 infection therapy, especially in infants and pregnant women, two subpopulations that have been poorly evaluated in the existing clinical trials. This narrative review is intended to offer insight into the existing literature on lactoferrin’s biological functions and protective effects against COVID-19, with a special focus on pregnant women and their infants.

## 1. Introduction

Severe acute respiratory syndrome coronavirus 2 (SARS-CoV-2) is a positive-sense, single-stranded, enveloped β-coronavirus with high genetic similarity with SARS-CoV, which caused a previous pandemic outbreak in 2002 [1,2,3]. Following the sudden appearance of several deadly cases of severe pneumonia of unknown origin in China at the end of 2019, SARS-CoV-2 was identified as the etiological agent of the coronavirus disease 19 (COVID-19) pandemic outbreak [1,2,4].

From a clinical point of view, COVID-19 shows very heterogeneous manifestations, ranging from nearly asymptomatic, flu-like conditions to severe symptoms that could evolve into interstitial pneumonia, acute respiratory distress syndrome (ARDS), multi-organ failure, and eventually, death [1,2,3,5].

COVID-19 is recognized as the greatest pandemic of the last 100 years, accounting for more than 20 million deaths worldwide from its emergence until mid-2023, when the World Health Organization (WHO) Emergency Committee declared the end of the public health emergency of international concern (PHEIC) status [6,7]. Although the newly developed COVID-19 vaccines and the implementation of targeted pharmacological treatments during the early phases of the disease strongly reduced the risk of experiencing the most severe COVID-19 manifestations [8], especially during the first waves of the pandemic, the lack of specific and effective therapeutic interventions to fight the disease fostered the research of new bioactive molecules, resulting in drug-repurposing approaches [9,10] as well as in the increasing popularity of over-the-counter nutritional supplements [10,11,12]. Among the latter, bovine lactoferrin quickly became one of the most popular, as it is well-tolerated, commercially available at low cost, and generally recognized as a safe (GRAS) nutritional supplement displaying a high homology and very similar bioactivity to the human protein [13,14,15].

Bovine lactoferrin has long been studied in different clinical contexts due to its high tolerability and safe profile, as testified by it being granted a US Food and Drug Administration (FDA) GRAS food additive and dietary supplement label in 2001 and European Food Safety Authority (EFSA) recognition as a novel food ingredient in 2012 [16,17]. Thanks to its antimicrobial and anti-inflammatory profile, lactoferrin has been proven to be effective in late-onset sepsis in premature, low-birth-weight neonates [18,19,20,21,22] as well as in the prevention and treatment of inflammatory intestinal diseases [23,24,25]. Furthermore, several randomized clinical trials also showed its efficacy in preventing upper and lower respiratory infections, as well as nosocomial and ventilator-associated infections in at-risk infants, children, and adults [24,26,27]. Finally, according to its iron-chelating ability, lactoferrin has been proven to be more effective than the standard ferrous sulfate supplementation in treating different kinds of anemia both in children and adults [28,29,30,31].

In this narrative review, we will summarize the available evidence about lactoferrin activity in infants and adults as well as in pregnant women. A literature search was conducted by screening the PubMed, Google Scholar, and Scopus repositories up to January 2024, using as keywords “lactoferrin”, “COVID-19”, “SARS-CoV-2”, “pregnancy”, “infants”, and “breast milk” alone or in combination. Furthermore, to avoid missing relevant papers dealing with the theme of interest, the references of the original articles and the related results suggested by the different repositories search engines were also considered. A further literature search was performed during the peer-review process, allowing the identification of new up-to-date important papers. Only papers published in English, where the full text was available, were used for this narrative review.

## 2. Biological Effects of Lactoferrin

Lactoferrin is a high-affinity, iron-binding cationic glycoprotein mainly found in mucosal secretions, especially in milk and colostrum. Moreover, it is also a major component of neutrophil granules, from which it is released following infective and/or inflammatory stimuli [1,3,13,32,33]. Lactoferrin and its digestion-derived products are known to exert many biological functions in a host’s defense against invading pathogens, displaying a wide range of antiviral, antimicrobial, and immunomodulatory activities [1,13,32,33] (Table 1).

Since the end of the last century, there have been several studies focused on lactoferrin’s ability to modulate both innate and adaptive immune responses [26,34,35,36]. It has been reported that lactoferrin concentration in biological fluids increases during inflammation, supporting its role in the host’s active defense against invading pathogens [37,38]. Considering its role in innate immunity, it is well accepted that this bioactive molecule exerts a direct antimicrobial action by limiting pathogen adhesion and proliferation and/or by killing microbes, thanks to its ability to sequester iron and destabilize microbial membranes, targeting, as an example, lipopolysaccharides (LPS) and lipoteichoic acid (LTA) [37,38,39,40,41,42]. In addition, it has been reported that lactoferrin is not only able to regulate immune cell proliferation, differentiation, and activation but also to modulate the production and release of both pro- and anti-inflammatory cytokines, thus playing a key role in regulating inflammatory responses [24,34,36,37,38,42,43].

A key feature allowing such a wide range of lactoferrin protective effects against infections is represented by its strong iron-binding ability [38,41,42]. By sequestering iron from biological fluids, lactoferrin reduces its availability to all the pathogens needing it for their survival, but also contributes to the restoration of correct iron homeostasis within the inflamed tissues, thus limiting the inflammation-related oxidative damage [35,36,38,40,43,44,45,46,47,48,49].

Furthermore, since the mid-1990s, lactoferrin antiviral action against both naked and enveloped viruses (e.g., cytomegalovirus, herpes simplex virus, human immunodeficiency virus, human hepatitis B and C viruses, rotavirus, echovirus 6) in both pediatric and adult populations has been reported by different research groups [37,46,50]. As no significant differences in the antiviral activity were found between highly or lowly iron-saturated lactoferrin, it is now well accepted that such ability to fight viral infections mainly relies on lactoferrin’s ability to prevent viral entry into host cells by blocking viral interaction with cellular receptors and/or by directly binding to virus particles [37,39,41,46,51,52,53,54,55]. Additionally, it has been demonstrated that lactoferrin could interfere with viral infections also at the post-entry level, by enhancing host inflammatory and interferon-mediated-antiviral responses [10,56,57,58,59,60].

Last but not least, lactoferrin has also gained interest in the context of microbiota homeostasis, as it has been demonstrated to be able to promote the growth of selected probiotic strains in both the gut and female reproductive tract, thus further contributing to boosting the host’s immune defense against pathogens [61,62,63,64].

## 3. Lactoferrin as Protection against COVID-19: Evidence from In Vitro Studies

Lactoferrin use as adjuvant therapy in COVID-19 has been hypothesized based on several in vitro pieces of evidence highlighting its ability to interfere with SARS-CoV and SARS-CoV-2 infection. The first evidence of the possible role of this widely available nutritional supplement in preventing coronavirus infection dates back to the SARS-CoV pandemic when Lang and coworkers [53] demonstrated lactoferrin’s ability to prevent SARS-CoV spike protein’s interaction with host cells by competing for the binding to the heparan sulfate proteoglycans on the host cell membranes, thus inhibiting the viral attachment phase.

As it is known that the SARS-CoV and SARS-CoV-2 viruses share not only a high sequence homology [1,2,4] but also the cell entry mechanism, based on their spike protein ability to use host cell membrane components such as angiotensin-converting enzyme 2 (ACE2) and heparan sulfate proteoglycans as entry receptor and co-receptors [4,57,65,66,67], several research groups tested the ability of lactoferrin to reduce or even prevent in vitro and in vivo SARS-CoV-2 infection.

Several researchers demonstrated that lactoferrin is able to inhibit SARS-CoV-2 viral replication, exhibiting multiple antiviral actions [10,65,68]. In silico docking experiments demonstrated that lactoferrin could directly bind SARS-CoV-2 spike protein [3,4,67], while in vitro experiments showed its competition with the virus for ACE2 [69] and heparan sulfate proteoglycan binding, thus inhibiting the very early infection stage [10,66,69]. Moreover, it has been demonstrated that this molecule is also able to enhance host immune responses, thus cooperating in limiting viral replication inside the host cells by activating interferon-dependent antiviral responses [10,32,57].

Furthermore, in addition to an effective antiviral activity, these in vitro studies also highlighted lactoferrin’s ability to downregulate IL-6 production and affect iron homeostasis, thus supporting its potential role in controlling COVID-19-associated cytokine storm and iron overload, the latter being two critical features of ARDS, one of the most severe COVID-19 complications [4,10,15,68,70,71].

## 4. Lactoferrin as Protection against COVID-19: Evidence from Clinical Studies

Such promising pre-clinical evidence fostered, therefore, the design of different clinical trials aimed to evaluate lactoferrin’s effectiveness in modifying disease evolution by acting as a preventive agent or as an adjuvant compound to be used in addition to the standard-of-care therapy [1,70]. To date, the results of only a few of the trials have been published (Table 2). It is worth noting that none of the trials was specifically focused on pregnant women and/or newborns; rather, all these studies were designed on adults, without any gender restriction, with female participants showing a median age above 40 years. Moreover, pregnancy and breastfeeding were among the exclusion criteria in at least two of the trials [72,73], while in the others, no data about pregnancy and/or breastfeeding were provided [14,74,75], thus impairing the possibility of drawing specific considerations about such specific subpopulations. Lastly, another important limitation of the existing literature is represented by the nature of the studies, as only two of them [73,75] were designed as controlled, randomized clinical trials, so it is possible that in the others, the observed results could be related, at least partially, to a lactoferrin-independent increase in iron bioavailability.

Although the early pilot studies reported a positive effect of lactoferrin supplementation in preventing SARS-CoV-2 infection [74] and in shortening the time to viral clearance as well as in accelerating clinical recovery [14,72], two other randomized clinical trials reported a lack of lactoferrin effectiveness in modifying disease evolution when administered as an adjuvant to the standard-of-care therapy. Even if in these trials, lactoferrin was not effective in modifying disease evolution, it showed an excellent safety and tolerability profile [73,75].

These different results in clinical practice could be explained by the different study designs adopted by the research groups, especially in terms of bovine lactoferrin formulation, the timing of initiation of the lactoferrin treatment, and disease severity, all aspects that could have a role in supporting the observed results.

First of all, several studies have highlighted that protected (i.e., microencapsulated or liposomal) vs. non-protected lactoferrin formulations have different bioavailability [70]. In this context, it should be considered that the intact molecule and its hydrolyzed fragments are known to display different biological activities, based on different mechanisms of action [13,76,77]. For this reason, a formulation able to protect lactoferrin from early digestion in the stomach could be of great importance in assuring a better absorption of the intact molecule at the intestinal level [70,78,79].

Secondly, it has been already reported that the mode of administration could affect lactoferrin bioactivity, as it is almost completely digested by gastric juices when taken during meals, while only a partial degradation is observed when the supplement is taken before meals [13,76]. Such an observation is of particular interest as it is well accepted that human intestine expresses a lactoferrin receptor [80,81,82], corresponding to intelectin-1 [81,83,84,85], which is able to internalize both human [80,81] and, albeit with lower efficiency, bovine lactoferrin [86,87], that is then supposed to enter the systemic circulation through the lymphatic system [13,35,88].

Thirdly, the time of lactoferrin administration during the course of COVID-19 disease could also have an important role in the observed heterogeneity in clinical trial results: while in the earlier studies, showing a clinical improvement, bovine lactoferrin was administered just following SARS-CoV-2 infection confirmation [14,72,74], in the latest study [75], the nutritional supplement administration started on the day of hospitalization, thus in patients with a more advanced disease, when it should be supposed that immune responses become independent from viral replication, as observed also in studies on antiviral treatments for COVID-19 [89,90].

Lastly, it should be considered that, when lactoferrin was administered during hospitalization, it was used as an add-on to the standard-of-care treatment, based on high doses of corticosteroids and heparin [75]; in this case, the anti-inflammatory activity of corticosteroids [91,92] could have, at least partially, masked lactoferrin’s immunomodulatory effects, while heparin could have similarly affected lactoferrin’s antiviral activity by competing with it for heparan sulfate proteoglycan binding [52,66,76].

Even though the limited and heterogeneous literature about lactoferrin supplementation in COVID-19 patients prevents the possibility of elaborating clear clinical guidelines on this topic, the good profile of safety and tolerability of this compound fosters new clinical trials designed and powered to evaluate lactoferrin effectiveness in improving COVID-19 clinical evolution, especially considering the high mutation rate of the SARS-CoV-2 viral agent and the proven antiviral activity of this nutritional supplement against the new emerging viral variants [30,65,70].

## 5. Lactoferrin and Pregnancy

Nowadays, it is well accepted that pregnancy represents a moment in women’s lives characterized by physiological changes affecting also the immune system, as testified by an increased maternal susceptibility and severity to certain infections, as well as by an associated increase in severe fetal outcomes [93,94]. For this reason, since the beginning of the pandemic, the effects of SARS-CoV-2 infection on both pregnant women and their infants have quickly become an important health concern worldwide, resulting in several national and transnational guidelines for pregnancy and delivery management.

To date, many studies have focused on SARS-CoV-2 infection during pregnancy and have highlighted that symptomatic pregnant women are at higher risk of severe COVID-19 evolution compared to non-pregnant females of the same age [95]. Moreover, it has been observed that SARS-CoV-2 infection during pregnancy is associated with an increased risk of developing pregnancy complications, such as miscarriage, premature birth, preterm rupture of membranes, pre-eclampsia, stillbirth, and fetal growth restriction [95,96]. Interestingly, many of these complications, as well as vaginal dysbiosis, could be successfully prevented by bovine lactoferrin supplementation, as reported by several researchers [62,63,97,98].

One of the most important SARS-CoV-2 targets during pregnancy is represented by the placenta, whose main function is to provide nutrients and support fetal development, as well as to represent a physical barrier to protect the developing embryo from invading pathogens as well as from maternal immune defenses [77,95,96,99].

It is worth noting that in pregnancy, lactoferrin represents one of the most important protective barriers at the maternal–fetal interface, as testified by its hormone-driven detection both in the endometrium and in the amniotic fluid. Moreover, the placenta is known to express ACE2 receptors, which are known to represent a prominent receptor for viral docking, thus accounting for the potential greater SARS-CoV-2 infection risk observed in pregnant women. Considering the wide range of beneficial biological actions of lactoferrin during pregnancy, it is not surprising that maternal–fetal interface lactoferrin represents an important mechanism of defense for both the mother and the fetus. In particular, endogenous lactoferrin could effectively downregulate ACE2 expression, thus reducing cellular receptors’ availability for viral entry, and also modulate pro-inflammatory response to COVID-19 [100,101].

According to the above-described lactoferrin protective mechanism, SARS-CoV-2 infection results in ACE2 placental downregulation, with consequent maternal hypertension and dysfunctional placental vascularization [95,102,103], as reported in several studies in which placentas from COVID-19 pregnant patients were examined, supporting the hypothesized relationship between the time of infection (reflecting the dynamic variation of ACE2 expression during gestation) and pregnancy outcomes [103,104,105,106].

SARS-CoV-2 infection during pregnancy also raised the question about the possibility of vertical transmission of the virus [97,107,108,109].

Despite its relationship with the MERS and SARS viruses, the COVID-19 etiological agent appears to be less lethal in pregnancy than other Coronaviruses (1% vs. 25%). In a recent review [110], most pregnant healthy women showed asymptomatic or low-grade symptoms, whereas women with complicated pregnancies (pre-eclampsia, advanced maternal age, hypertension, gestational diabetes) were at a higher risk of developing severe disease than the general population.

The U.S. CDC’s COVID-19 surveillance system provided data regarding pregnancy and COVID-19 that require attention. Compared with nonpregnant women, pregnant patients were 3 times more likely to be admitted to an intensive care unit (ICU: 10% vs. 3%) and 1.7 times more likely to die (1.5 vs. 1.2 per 1000 cases) [111]. Similar results were disclosed by a multicenter European-based study comparing pregnant and nonpregnant women matched for age, BMI, and comorbidities [112].

Pregnancy adverse outcomes are known to be associated with hyperinflammation and altered iron homeostasis [30,31] as well as to infections of the placental and/or amniotic environment [113,114,115,116]. Physiologically healthy fetal development is assured by the mother’s defense system, in which lactoferrin plays a pivotal role. It is extensively secreted by the cervix and syncytiotrophoblasts, as it can be detected in amniotic fluid from the 20th week of gestation. The fetus absorbs amniotic lactoferrin by swallowing and a progressive build-up during pregnancy is observed [117], and its concentration rises markedly from the 32nd gestational week onwards [114,116]. This pleiotropic molecule has been described to be present also in the female genital system [62], where it is mainly produced by the epithelium lining the uterus and vagina under hormonal control and by circulating neutrophils recruited in case of infection. Lactoferrin presence in placenta and amniotic fluid is involved in the prevention of pregnancy complications thanks to its anti-infective and anti-inflammatory properties and thus, it is conceivable that it could play a role also in preventing SARS-CoV-2 vertical transmission and COVID-19 pregnancy negative outcomes [114,115,118].

## 6. Lactoferrin and Infants

COVID-19 disease in infants is not as prevalent as in adults, and the infections are usually asymptomatic or mildly symptomatic. In neonates, the disease’s clinical manifestations are different from those observed in older children and adults, and, importantly, the prognosis is usually good [102,119,120]. Nevertheless, little is known about the possibility for the infected neonates to experience long-term COVID-19 consequences and further studies focused on perinatal and antenatal viral infection’s long-term effects on child development are warranted [120].

Given the reported low rate of vertical transmission, the occurrence of neonatal infections mainly depends on postpartum events, such as direct contact with contaminated fomites or exposure to maternal infectious respiratory droplets and/or secretions [95,119,121].

The emergence of the COVID-19 pandemic resulted in several changes in perinatal care management with the aim of minimizing SARS-CoV-2 mother-to-child transmission as well as viral spread in hospitals, despite the fact that since the very first moments of the pandemic, the World Health Organization endorsed early continuous skin-to-skin contact and breastfeeding [122,123].

Since the 1990s, the WHO and the United Nations Children’s Fund (UNICEF) have promoted breastfeeding, as breast milk is known to supply both essential nutrients and bioactive molecules fundamental to supporting immune system development [122,124,125]. The well-known role of breast milk in supporting neonatal immune system maturation along with the observed low rate of SARS-CoV-2 infection in neonates account for the increasing number of studies focused on COVID-19 during breastfeeding.

It is noteworthy that SARS-CoV-2 RNA detection in human milk from COVID-19 patients is uncommon. Viral RNA has been detected in breast milk only sporadically and none of the positive samples showed live, active, and replication-competent virus, accounting for the lack of reports confirming viral transmission via breastfeeding [121,123,126,127]. Furthermore, several researchers successfully detected anti-SARS-CoV-2 antibodies (i.e., IgA, IgM, IgG, at least one of these types, if not all of them) in breast milk obtained both from COVID-19-infected or vaccinated breastfeeding women, further sustaining the protective role of breast milk toward newborn infection [120,128,129,130,131]. Lastly, it should be remembered that human milk also contains other bioactive compounds with immunomodulatory activities, such as micro RNAs, which are known to play an important role in newborn immune system development and protection from different pathological conditions [132,133].

Lactoferrin is one of the most abundant proteins of human breast milk; its concentration is maximal in colostrum and then decreases in mature milk, with a reduction trend that reflects the stage of lactation [134,135,136,137]. It is well accepted that lactoferrin and its derivatives, such as lactoferricin and lactoferrampin, display a strong antimicrobial effect against both viral and bacterial agents, thus contributing to the protection of the newborn from infections [25,29,50,54,55,59,138]. Accordingly, infant formula fortification with exogenous lactoferrin has been suggested for a long time [139], and a few clinical trials showed bovine lactoferrin supplementation’s effectiveness in preventing respiratory and intestinal infections in infants [139,140,141] as well as in preventing severe disease manifestations such as necrotizing enterocolitis (NEC) and sepsis in preterm infants [20,21,139,142]. Such a prevalence of lactoferrin beneficial effects in infants compared to the adult population can be explained by the immature digestive system of the newborn. It is known that gastric and pancreatic enzymes are present in lower amounts and with reduced activity in newborns, accounting for reduced protein hydrolysis, as confirmed in vitro by a dynamic digestion system simulating the infant digestion process. Such an incomplete lactoferrin digestion allows its intestinal absorption in the intact form, which can reach unaltered the bloodstream, which accounts for its distribution toward the different tissues expressing its specific receptors. This evidence supports the hypothesis that lactoferrin in infants is not completely digested, thus maintaining its physiological activities and supporting its protective role toward the newborn [143].

In this context, it is conceivable that in addition to maternal antibodies, lactoferrin and other milk proteins with antiviral activity could also play a key role in protecting newborns from COVID-19 infection [21,126,128,129].

Such a hypothesis is supported by the observation that lactoferrin levels vary not only following a lactation-stage-driven trend but also according to SARS-CoV-2 infection history. It has been observed that lactoferrin levels in colostrum (which represents the first nutrient available for the infant just after delivery) from women with an active infection at the time of delivery were higher compared to those of healthy subjects or subjects who had recovered from the infection [144]. Conversely, when considering transition and mature milk, lactoferrin levels were lower in milk of symptomatic COVID-19 mothers compared to asymptomatic ones and healthy controls [126]. While it is known that lactoferrin production is upregulated during coronavirus infection, its high levels in colostrum and its subsequent reduction in symptomatic patients’ milk could be due to consumption during the antiviral response, thus accounting for the low incidence of infections in neonates even in the presence of SARS-CoV-2-positive mothers [120,126].

Furthermore, the antiviral activity of human-milk-derived lactoferrin was reported by Lai and coworkers [145], who demonstrated that several components of breast milk (which included lactoferrin) were able to reduce SARS-CoV-2 pseudovirus as well as transcription- and replication-competent SARS-CoV-2-virus-like particle infection in different in vitro models. These results are even more interesting given that the milk samples used to perform fraction isolation and antiviral activity tests were collected before the COVID-19 pandemic, thus excluding any potential role of maternal anti-SARS-CoV-2 antibodies in the observed results. Importantly, they also demonstrated that breastmilk-derived lactoferrin, as well as mucin-1 and α-lactalbumin (the other two main antiviral components of breastmilk), showed a high antiviral activity towards different viral variants (e.g., beta, gamma, and kappa), thus supporting the hypothesized protective role of breastfeeding.

In conclusion, the whole of the aforementioned findings (absence of replicating virus in breast milk from COVID-19-positive lactating mothers with low disease activity, decreased lactoferrin levels in milk of infected mothers, absence of proven viral mother-to-child transmission via breastfeeding) suggests that the lactoferrin naturally occurring in human breast milk is consumed in the attempt to combat SARS-CoV-2 infection, thus decreasing viral load and providing a limited viral shedding in milk. Moreover, the high lactoferrin levels detected in the colostrum of mothers with active infection could represent another way adopted by nature to protect newborns from direct or indirect viral transmission. The available evidence in this field thus supports once more the key role of breastfeeding in protecting infants from postnatal infections, including COVID-19.

## 7. Conclusions

Lactoferrin is a glycoprotein with pleiotropic antimicrobial as well as immune-modulating properties produced by different cell populations. In humans, infections induce a sustained release of this bioactive molecule from neutrophils’ secondary granules, thus representing a first-line defense against invading pathogens. Furthermore, it is produced by epithelial cells, contributing to limiting microbial spread at the mucosal level. Interestingly, in females, lactoferrin undergoes also a hormone-regulated production by the epithelium lining the genital system, thus accounting for the protection of the developing fetus against antenatal infections. Finally, lactoferrin is a component of human colostrum and milk, playing a key role in supporting infant defense and immune system maturation, by exerting a prebiotic as well as a probiotic function.

Although the WHO declared that COVID-19 is no longer a PHEIC, it still represents an important health issue, thus fostering world nations to move from emergency management of the disease to a new phase, in which SARS-CoV-2 should be managed alongside other endemic infections. Although recent developments in COVID-19 vaccines and early therapeutic approaches have strongly reduced the risk of severe disease manifestations, the limited number of marketed specific and effective therapies resulted in the increased popularity of over-the-counter nutritional supplements as therapeutic adjuvants or preventive agents, especially in resource-limited settings. Among those, bovine lactoferrin has been very popular, thanks to its high tolerability and safe profile.

To date, lactoferrin’s effectiveness in improving COVID-19 clinical outcomes has been evaluated in several cohort studies in the adult general population with conflicting results: in early studies involving asymptomatic or mildly symptomatic patients, lactoferrin administration reduced the time to negativization and/or prevented the infection, while in recent studies, involving moderate and severe hospitalized patients, it failed to improve disease course.

A major limitation of our review is represented by the lack, to the best of our knowledge, of studies specifically focused on lactoferrin’s protective action against SARS-CoV-2 in pregnant women and their newborns. Considering the available evidence about the low mother-to-child transmission rate as well as the low frequency of COVID-19 in neonates, further dedicated studies aimed at investigating lactoferrin’s protective role also in these specific populations will be of great interest.

To date, only a few clinical studies dealing with lactoferrin’s role in COVID-19 have been completed and published; furthermore, it should be considered that among these studies, only two were randomized controlled clinical trials, while the others were only observational in nature. Moreover, several promising results come from in vitro evaluations, thus further complicating the extrapolation of strong clinical evidence. Considering these intrinsic limitations of the available literature, it is clear that these limited and heterogeneous data do not warrant the creation of clinical guidelines on this topic for either adult or infant populations. Nevertheless, whilst breastfeeding has not been demonstrated to assure any specific protection to infants from SARS-CoV-2, breastfeeding should be supported in a general context to imbue newborns with broad protection against infections and illnesses, taking advantage of both the antimicrobial milk components, such as lactoferrin, and the mother-derived specific antibodies.

## Figures and Tables

**Table 1:** Lactoferrin biological activities.

Biological Activity	Main Mechanism Involved
Regulation of iron homeostasis	Binding of two Fe^3+^ ion/molecule with high affinity, assuring the low iron bioavailability (10^−18^ M) typical of a healthy state
Antibacterial activity	Bacteriostatic and bactericidal activities depending on the considered microorganism
Antiviral activity	Inhibition of enveloped and naked virus binding and entry into the host cells
Immunomodulatory activity	Stimulation of innate immune responses and stimulation of immature B and T cells maturation towards antigen-presenting cells and T helper lymphocytes, respectively
Promotion of microbiota diversity	Promotion of selected probiotic strains’ growth over pathogenic ones in both gut and female reproductive system bacterial flora
Anti-inflammatory activity	Downregulation of pro-inflammatory cytokines (i.e., IL6, IL8, IL1β, TNFα) expression Upregulation of anti-inflammatory cytokines (i.e., IL4, IL10) expression Reactive oxygen species scavenging

**Table 2:** Summary of the published results from clinical studies dealing with lactoferrin effectiveness against COVID-19.

Study Type	Mani Findings	Country	References
Retrospective study	Reduction in the time to negativization in ambulatory-treated asymptomatic, paucisymptomatic, and moderate symptomatic patients	Italy	[14]
Randomized, parallel arm, interventional, open-label clinical trial	Reduction in the time to negativization and improvement in COVID-19 symptoms in asymptomatic and mild-to-moderate patients (both hospitalized and home-treated)	Italy	[72]
Prospective, observational study	Improvement in COVID-19 symptoms in home-treated patients	Spain	[74]
Randomized, double-blind, multicenter, placebo-controlled, parallel-arm clinical trial	No additional benefits to the standard-of-care therapy in hospitalized patients	Italy	[75]
Randomized, prospective, interventional clinical trial	No additional benefits to the standard-of-care therapy in hospitalized patients	Egypt	[73]
